# Abrogation of Rbpj Attenuates Experimental Autoimmune Uveoretinitis by Inhibiting IL-22-Producing CD4^+^ T Cells

**DOI:** 10.1371/journal.pone.0089266

**Published:** 2014-02-28

**Authors:** Zaied Ahmed Bhuyan, Michihito Asanoma, Akiko Iwata, Chieko Ishifune, Yoichi Maekawa, Mitsuo Shimada, Koji Yasutomo

**Affiliations:** 1 Department of Immunology & Parasitology, Institute of Health Biosciences, The University of Tokushima Graduate School, Tokushima, Japan; 2 Digestive and Pediatric Surgery, Institute of Health Biosciences, The University of Tokushima Graduate School, Tokushima, Japan; 3 Ophthalmology and Visual Neuroscience, Institute of Health Biosciences, The University of Tokushima Graduate School, Tokushima, Japan; New York University, United States of America

## Abstract

Experimental autoimmune uveoretinitis (EAU) is an organ-specific T cell-mediated disease induced by immunizing mice with interphotoreceptor retinoid binding protein (IRBP). Autoaggressive CD4^+^ T cells are the major pathogenic population for EAU. We investigated the contribution of Notch signaling in T cells to EAU pathogenesis because Notch signaling regulates various aspects of CD4^+^ T cell functions. Rbpj is required for Notch signaling, and *Rbpj* deficiency in T cells inhibited EAU disease severity. The amelioration of EAU in T cell-specific Rbpj-deficient mice correlated with low levels of IL-22 production from CD4^+^ T cells, although IRBP-specific CD4^+^ T cell proliferation and Th17 differentiation were unaffected. Administration of recombinant IL-22 during the late phase, but not the early phase, of EAU increased EAU clinical scores in T cell-specific Rbpj-deficient mice. Notch inhibition in mice immunized with IRBP with a γ-secretase inhibitor (GSI) suppressed EAU progression, even when GSI was administered as late as 13 days after IRBP immunization. Our data demonstrate that Rbpj/Notch-mediated IL-22 production in T cells has a key pathological role in the late phase of EAU, and suggest that Notch blockade might be a useful therapeutic approach for treating EAU.

## Introduction

Experimental autoimmune uveoretinitis (EAU) is an organ-specific, T cell-mediated disease initiated by immunizing mice with retinal antigens or their fragments [1] [2] [3] [4]. EAU represents a breakdown in tolerance to immunologically privileged retinal antigens, such as interphotoreceptor retinoid binding protein (IRBP) and arrestin, which function in the visual cycle. EAU is a model for human ocular diseases including Behcet's disease, Vogt-Koyanagi-Harada syndrome, and Birdshot retinochoroidopathy [1] [4]. CD4^+^ T cells are crucial for EAU development, which is supported by the finding that EAU can be elicited by adoptive transfer of retinal-specific CD4^+^ T cells [5]. Although Th1 cells are activated in EAU, it has been reported that IL-12 down regulates EAU, and treatment of mice with EAU with anti-IFN-γ antibodies aggravated the disease [6] [7]. Subsequent reports have demonstrated the importance of Th17 in EAU pathogenesis [8] [9]. In therapeutic settings, it is essential to identify which T cells or which cytokines are crucial for each phase during EAU progression or human autoimmune uveoretinitis.

Notch is an evolutionarily conserved molecule that controls cell fate decisions in a variety of cells [10]. Notch molecules are cleaved in their transmembrane region by γ-secretase through interaction with Notch ligands, after which the intracellular domain translocates into the nucleus [10]. We and other groups have demonstrated that Notch signaling controls CD4^+^ T cell effector functions [11] [12] [13] [14] [15]. Moreover, it has been reported that inhibition of γ-secretase or Delta-like 4 (Dll4) blocked the development of experimental autoimmune encephalomyelitis [16] [17] [18], suggesting that Notch signaling in T cells is involved in the progression of autoimmune responses. Although anti-Dll4 antibody ameliorates EAU [19], the roles of Notch signaling in T cells for EAU progression remain unclear.

These earlier reports led us to investigate the role of Notch signaling in the development of EAU. In this study, we show that T cell-specific *Rbpj*-deficient mice are resistant to EAU, and that this dramatic outcome derives from low IL-22 production by CD4^+^ T cells without affecting T cell priming and Th17 differentiation. Treatment of mice with a γ-secretase inhibitor (GSI) attenuated the progression of EAU. The resistance of *Rbpj* conditional knockout mice to developing EAU and the successful treatment of EAU by GSI suggest that the Notch pathway is a potential therapeutic target in inflammatory ocular disease.

## Materials and Methods

### Animals

Six- to 8-wk-old C57BL/6 mice were purchased from Japan SLC (Hamamatsu, Japan). *Rbpj* ^flox/flox^ mice crossed CD4-*Cre* transgenic mice or E8I-*Cre* transgenic mice are described elsewhere [12] [20]. All mice were housed under specific pathogen-free conditions in the Animal Research Center of the University of Tokushima. Animal care and use was in compliance with institutional guidelines and was approved by the Animal Research Committee of the University of Tokushima.

### Antibodies and flow cytometry

Monoclonal antibodies specific for mouse CD4 (GK1.5) or CD8 (53-6.7) were purchased from BioLegends (San Diego, CA, USA). Flow cytometry data were acquired on a FACSCaliber (BD Biosciences, CA, USA) and CellQuest (BD Biosciences) software was used for analysis.

### Induction of EAU

Mice were immunized subcutaneously with 50 µg of IRBP emulsified with CFA (St. Louis, MO) that had been supplemented with *M. tuberculosis* strain H37RA (Difco, Detroit, MI) to a final IRBP concentration of 2.5 mg/ml. Concurrent with immunization, 0.5 µg of pertussis toxin was injected intraperitoneally. The severity of eye disease was evaluated by fundoscopic examination. Mice were anesthetized and their pupils dilated. The fundus was observed using a stereoscopic microscope. Disease severity was scored on a scale of 0 (no disease) to 4 (maximum disease) depending on the degree of inflammation and retinal damage using established criteria.

### Treatment of mice with GSI or rIL-22

Mice immunized with IRBP were treated with GSI LY-411575 (Stemgent, San Diego, CA) (5 mg/kg/d, dissolved in DMSO) at 2-day intervals that began on the day of IRBP immunization. Recombinant IL-22 (Peprotec) (2 µg/dose) was administered to mice that had been immunized with IRBP at 3-day intervals that began one day after IRBP immunization and stopped 13 days after immunization, began 14 days after immunization and continued throughout the entire experiment, or began one day after IRBP immunization and continued throughout the entire experiment.

### T cell proliferation assay

Draining lymph nodes were collected and pooled within each group. For purification of total CD4^+^ cells, lymph node cells were incubated with anti-B220 (RA3-6B2), anti-CD32/16 (2.4G2), anti-CD11b (M1/70) and anti-CD8 (53-6.7) mAbs (BioLegends) followed by incubation with anti-rat IgG-coated Dynabeads (Dynal Inc.). CD4^+^ T cells were further purified by magnetic separation using biotin-conjugated anti-CD4 and streptavidin microbeads (Miltenyi Biotech). CD4^+^ T cells were cultured with irradiated spleen cells with 0.1, 1 or 10 µg/ml IRBP in 96-well round-bottom plates at a concentration of 5×10^5^ cells/well in RPMI 1640 supplemented with 2-ME, glutamine, nonessential amino acids, sodium pyruvate, and antibiotics, and 10% fetal bovine serum. For T cell proliferation assays, the cultures were incubated for 72 h and were pulsed with [^3^H]-thymidine (1.0 µCi/10 µl/well) for the last 6 h.

### Cytokine assay

Purified CD4^+^ T cells were cultured with irradiated spleen cells in 96-well flat-bottom plates at a concentration of 1×10^6^ cells/well in 0.2 ml of medium. Cells were stimulated with 50 µg/ml of IRBP. ELISAs for IFN-γ, IL-17 and IL-22 were performed on 72-h supernatants using ELISA kits from eBiosciences or R&D systems, respectively.

### PCR

Total RNA was extracted with Trizol (Invitrogen). After reverse-transcription with Omniscript RT Kits (Qiagen), cDNA was amplified by primers for *Hes1*, 5′- GTGGGTCCTAACGCAGTGTC-3′ and R5′-GTCAGAAGAGAGAGGTGGGCTA-3′; For real-time PCR, after reverse-transcription with Omniscript RT Kits (Qiagen), SYBR premix Ex Taq II (Takara Bio) was used for quantitative PCR. All data were normalized to HPRT (hypoxanthine-guanine phosphoribosyl transferase) and were presented as fold increase relative to the background value. The sequences for primers were as follows: 5′-CACGGCCCTGGTTCTCAT-3′; reverse primer, 5′-CAGATGTTCCACTCTCCTCTTCTCT-3′, *Actin*, 5′-TGGGAATGGGTCAGAAGGACT-3′ and 5′-TTTCACGGTTGGCCTTAGGGT-3′

### Statistical analysis

Student's *t*-test was performed for statistical analyses.

## Results

### Rbpj deficiency in T cells suppresses EAU development

**Figure 1:**
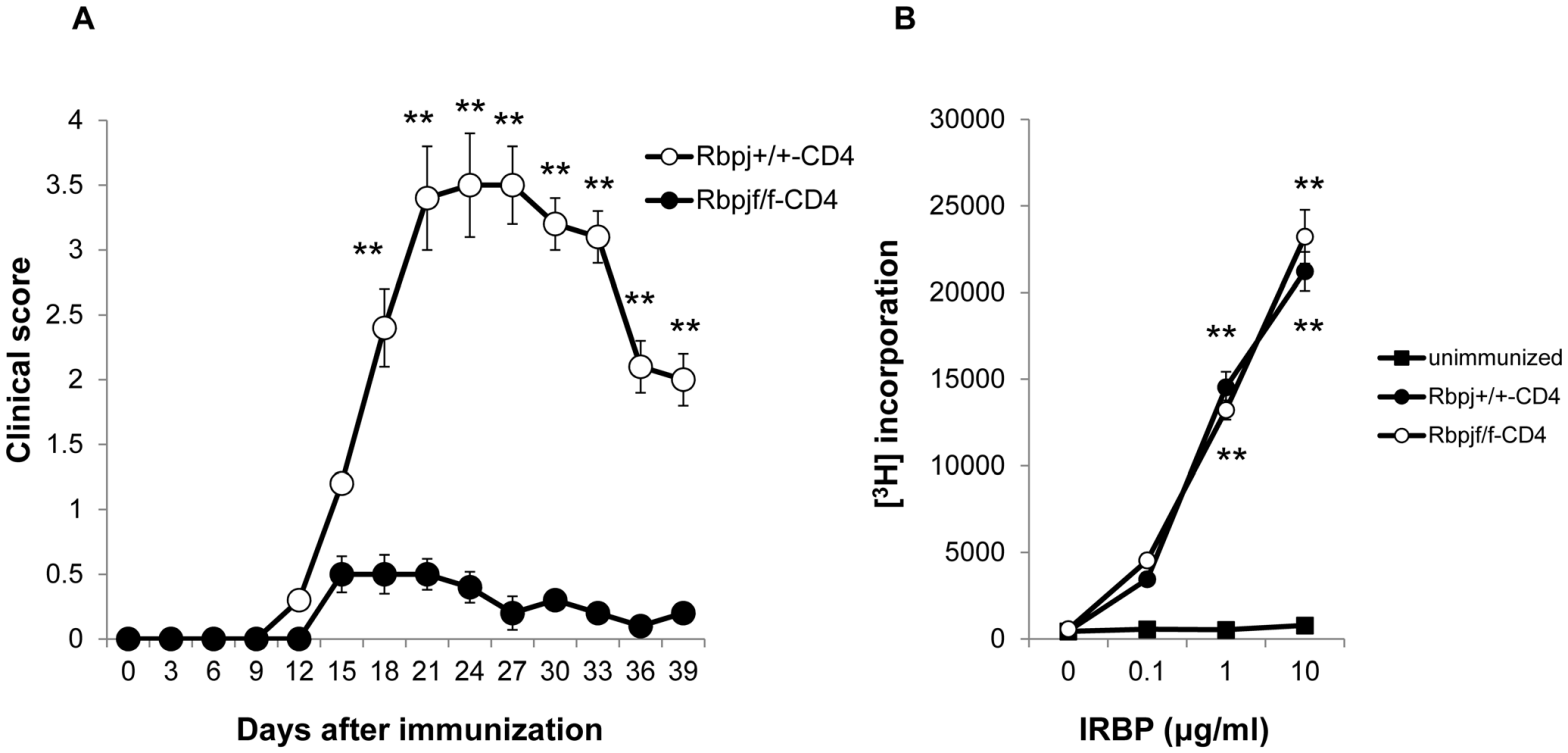
*Rbpj* deficiency in T cells ameliorates the induction of EAU. (A) Rbpj^f/f^-CD4 and Rbpj^+/+^-CD4 mice were immunized with IRBP to induce EAU and EAU clinical scores were evaluated until day 39. The data are the mean ± SD with five mice per group. **p<0.01. The score for each mouse was the average for both eyes. (B) Purified CD4^+^ T cells from Rbpj^f/f^-CD4 and Rbpj^+/+^-CD4 mice immunized with IRBP were stimulated with increasing doses of IRBP protein and their proliferative responses to antigen were measured by [^3^H] thymidine uptake. The results shown are the mean ± SD for triplicate cultures. **p<0.01. The data in this Figure are representative of three independent experiments.

Rbpj is crucial for Notch signaling [21]. In order to evaluate if Notch signaling in T cells is involved in EAU development, we attempted to induce EAU in *Rbpj* ^flox/flox^ mice crossed with CD4-*Cre* transgenic mice (Rbpj^f/f^-CD4) that do not express Rbpj in mature CD4^+^ and CD8^+^ T cells [12]. *Rbpj* ^+/+^ mice crossed with CD4-*Cre* transgenic mice (Rbpj^+/+^-CD4) were used as controls. These two strains were immunized with interphotoreceptor retinal binding protein (IRBP) using the standard uveitogenic protocol described in the Material and Methods. Rbpj^f/f^-CD4 mice exhibited lower EAU clinical scores compared to Rbpj^+/+^-CD4 mice over the course of disease progression (Fig. 1A). To test if the initial priming of T cells is impaired in Rbpj^f/f^-CD4 mice, CD4^+^ T cell proliferation was examined seven days after immunization with IRBP. CD4^+^ T cells from Rbpj^f/f^-CD4 mice exhibited similar proliferative responses against IRBP to those of Rbpj^+/+^-CD4 mice (Fig. 1B). These data indicate that deficiency of Rbpj in T cells decreases the susceptibility for EAU and suggest that the initial CD4^+^ T cell priming or proliferation against IRBP is not affected by *Rbpj* deficiency.

### Rbpj deficiency decreases IL-22 production in CD4^+^ T cells after induction of EAU

**Figure 2:**
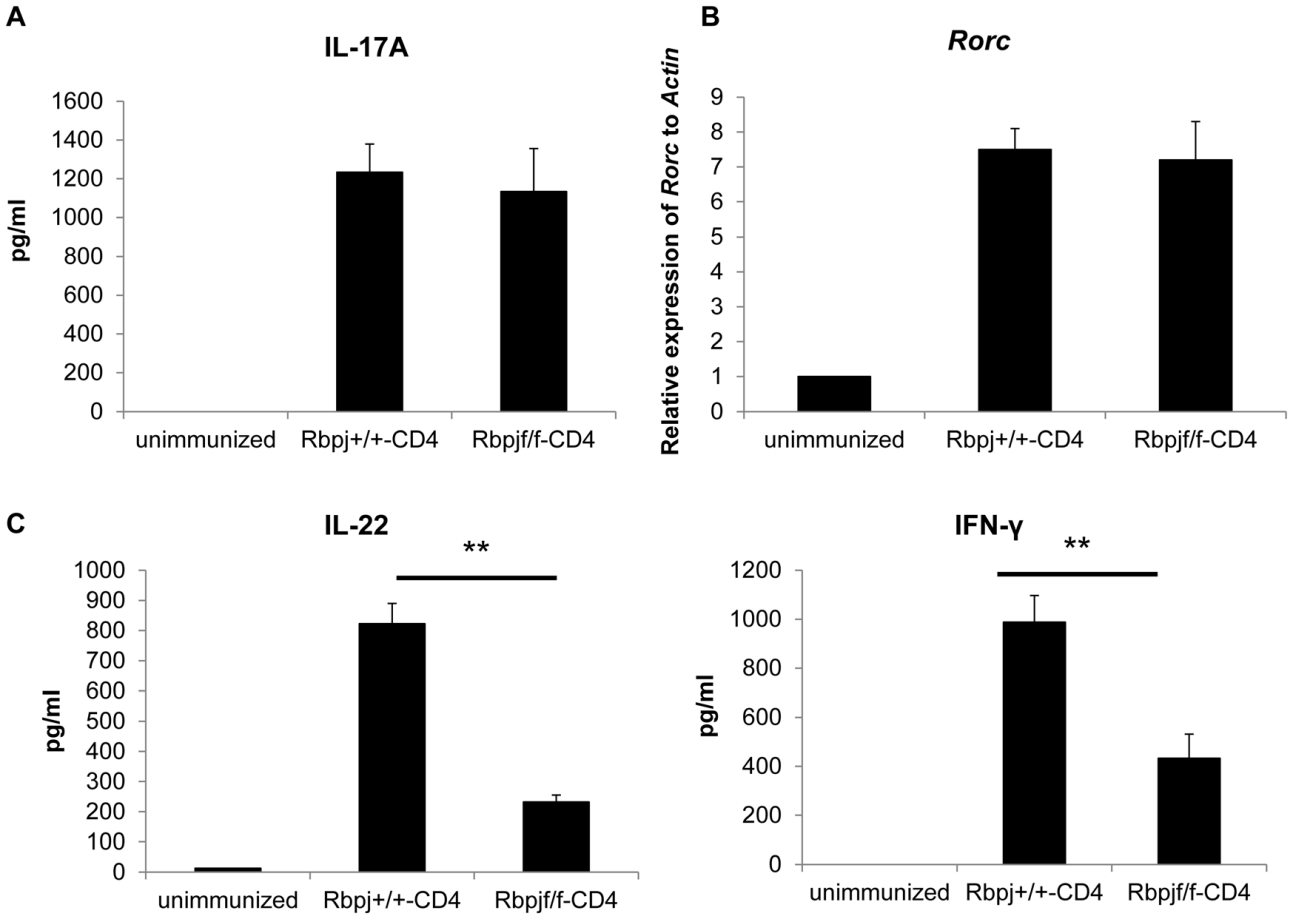
*Rbpj* deficiency in T cells reduces of IL-22 production. Rbpj^f/f^-CD4 and Rbpj^+/+^-CD4 mice were immunized with IRBP and CD4^+^ T cells were purified from draining lymph nodes 7 days after immunization. Purified CD4^+^ T cells were cultured with irradiated spleen cells from B6 mice in the presence of IRBP protein (50 µg/ml) for 3 days. (A) IL-17 in culture supernatants was evaluated by ELISA. (B) mRNA was purified from CD4^+^ T cells 7 days after immunization and the relative levels of *Rorc* against *Actin* were evaluated by real-time PCR. (C) IL-22 and IFN-γ in culture supernatants 72 hours after stimulation of purified CD4^+^ T cells from Rbpj^f/f^-CD4 and Rbpj^+/+^-CD4 mice immunized with IRBP (7 days previously) was evaluated by ELISA. The data are the mean ± SD from triplicate cultures. **p<0.01. The data in this Figure are representative of three independent experiments.

We next examined whether Rbpj affects the differentiation of IL-17- (Th17) producing CD4^+^ T cells because that T cell population is involved in EAU development [8] [9] [22]. Either Rbpj^f/f^-CD4 or Rbpj^+/+^-CD4 mice were immunized with an uveitogenic protocol with IRBP. On day 7, IL-17 production from CD4^+^ T cells stimulated with IRBP was analyzed by ELISA. IL-17 production was comparable between the 2 groups on day 7 (Fig. 2A). As Th17 differentiation requires the transcription factor Rorc, we investigated the mRNA expression of *Rorc* in CD4^+^ T cells from Rbpj^f/f^-CD4 and Rbpj^+/+^-CD4 mice 7 days after IRBP immunization. We found that the mRNA expression of *Rorc* is comparable between CD4^+^ T cells from Rbpj^f/f^-CD4 and Rbpj^+/+^-CD4 mice (Fig. 2B). These data suggest that Th17 differentiation is not affected by *Rbpj* deficiency in T cells at least 7 days after IRBP immunization.

As we have previously reported that *Rbpj* deficiency in T cells reduces IL-22 production from T cells [23], we investigated the production of IL-22 in CD4^+^ T cells from Rbpj^f/f^-CD4 mice 7 days after immunization by IRBP. IL-22 production by CD4^+^ T cells from Rbpj^f/f^-CD4 mice was much less than from control mice (Fig. 2C). The INF-γ production was also impaired by *Rbpj* deficiency (Fig. 2C). These data demonstrate that Rbpj is required for the production of IL-22 from CD4^+^ T cells while not affecting the differentiation of Th17 cells in the EAU model.

### Notch signaling in CD8^+^ T cells is not involved in the susceptibility for EAU

**Figure 3:**
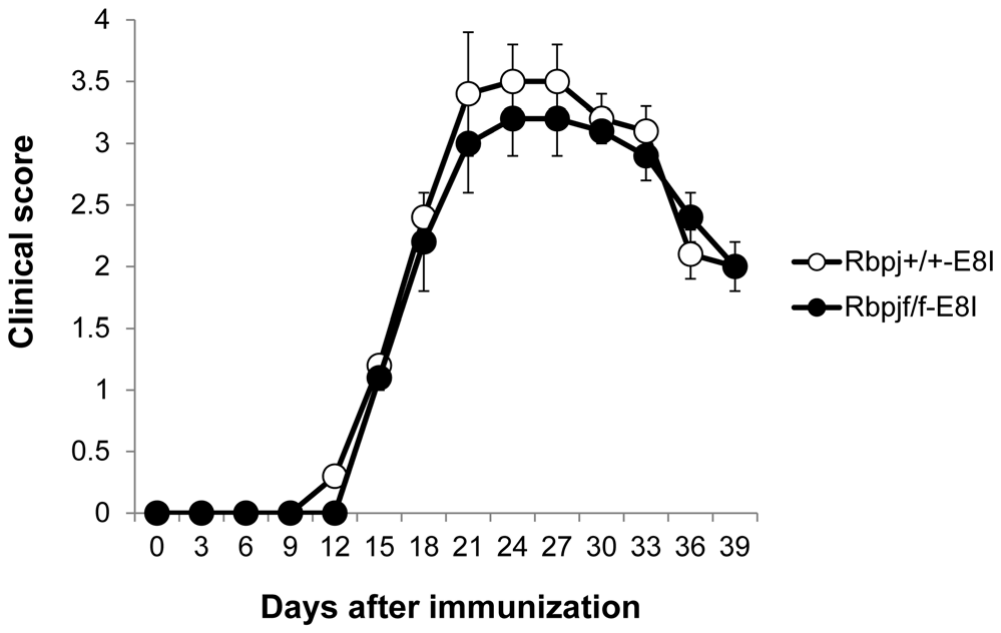
*Rbpj* deficiency in CD8^+^ T cells does not alter EAU susceptibility. Rbpj^f/f^-E8I and Rbpj^+/+^-E8I mice were immunized with IRBP to induce EAU and EAU clinical scores were evaluated until day 39. The data are the mean ± SD with five mice per group. The score for each mouse was the average for both eyes. The data in this Figure are representative of three independent experiments.

Rbpj^f/f^-CD4 mice lack *Rbpj* in both mature CD4^+^ and CD8^+^ T cells. Although previous studies have not revealed a clear contribution of CD8^+^ T cells to EAU progression [24], we investigated whether Rbpj deficiency in only mature CD8^+^ T cells impacts EAU susceptibility. *Rbpj* ^flox/flox^ mice were crossed with E8I-*Cre* transgenic mice (Rbpj^f/f^-E8I) in which *Rbpj* is deleted in mature CD8^+^ but not CD4^+^ T cells [20]. Rbpj^f/f^-E8I and Rbpj^+/+^-E8I mice were immunized with IRBP and EAU development was monitored. We observed that Rbpj^f/f^-E8I mice developed EAU similar to controls over the course of the disease (Fig. 3). Therefore, Rbpj in mature CD8^+^ T cells does not play a major role in the development of EAU.

### IL-22 contributes to the progression of EAU

**Figure 4:**
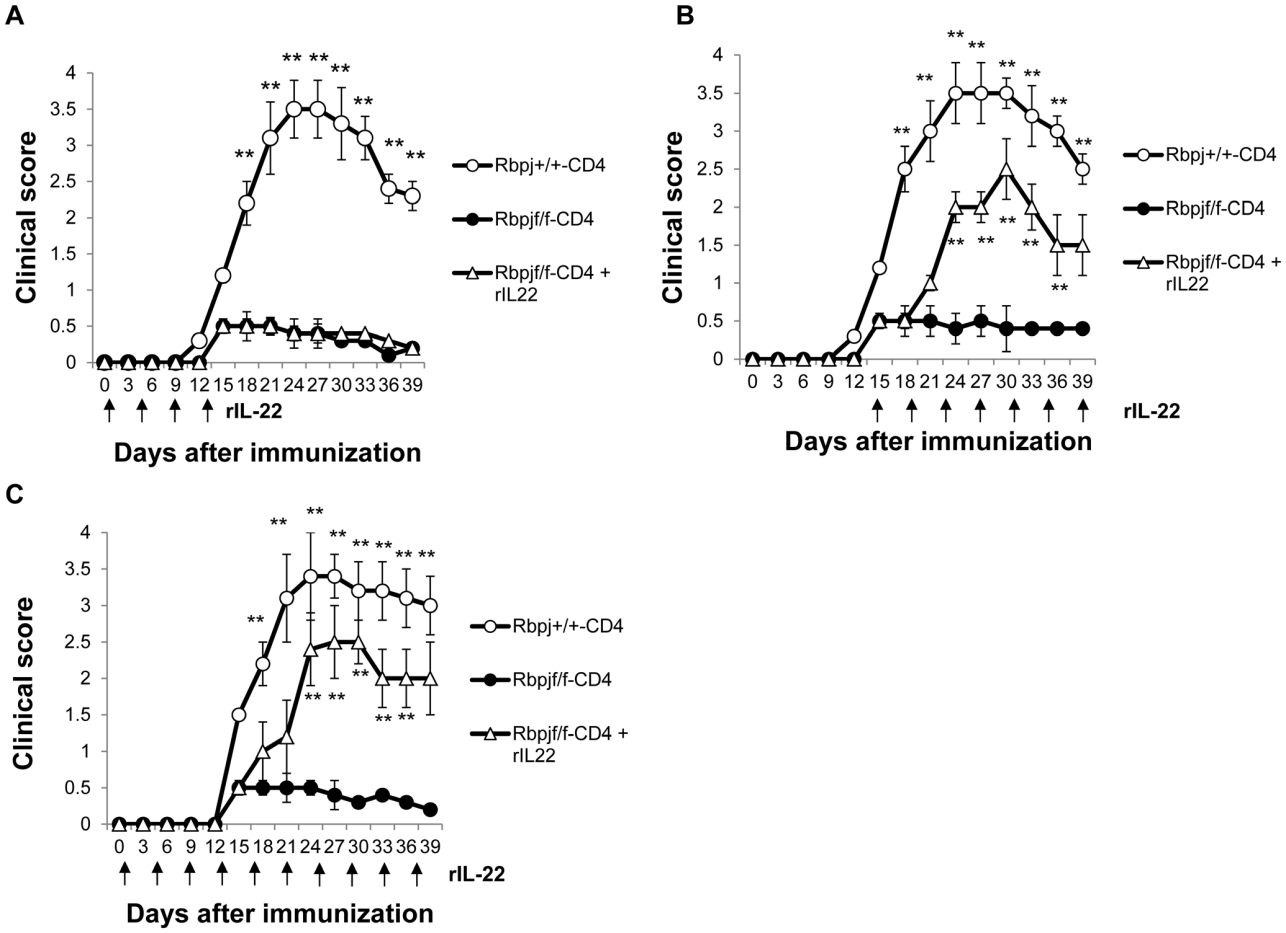
IL-22 is involved in the progression of late-phase EAU. Rbpj^f/f^-CD4 and Rbpj^+/+^-CD4 mice were immunized with IRBP to induce EAU and EAU clinical scores were evaluated until day 39. rIL-22 was administered to the mice according to the following schedules; the day of IRBP immunization is day 0. (A) 3-day intervals (from day 1 to day 13), (B) 3-day intervals (from day 14 to day 39), (C) 3-day intervals (from day 1 to day 39). The data are the mean ± SD with five mice per group. **p<0.01. The data in this Figure are representative of three independent experiments.

We next sought to investigate if IL-22 is involved in the progression of EAU. Recombinant IL-22 (rIL-22) was administered to Rbpj^f/f^-CD4 mice that had been immunized with IRBP at 3-day intervals beginning one day after IRBP immunization and ceased 13 days after immunization, beginning 14 days after immunization and continued throughout the course of the experiment, or beginning one day after IRBP immunization and continued over the course of the experiment. The administration of rIL-22 during the early phase of EAU in Rbpj^f/f^-CD4 mice did not change the clinical scores of EAU compared to that of Rbpj^+/+^-CD4 mice (Fig. 4A). In contrast, the administration of rIL-22 during the late phase of EAU aggravated EAU clinical scores (Fig. 4B), with measurable differences being observed 6 days after the initial rIL-22 administration. The rIL-22 administration during the late phase of EAU did not cause chronic EAU because mice that received rIL-22 recovered from EAU at day 60 (data not shown). The administration of rIL-22 over the course of EAU also aggravated EAU clinical scores, although it still did not reach the scores of Rbpj^+/+^-CD4 mice (Fig. 4C). These data demonstrate that Notch-mediated IL-22 from T cells is crucial for the development of late phase EAU progression, rather than the early phase.

### Treatment with GSI during later disease stages inhibited EAU

**Figure 5:**
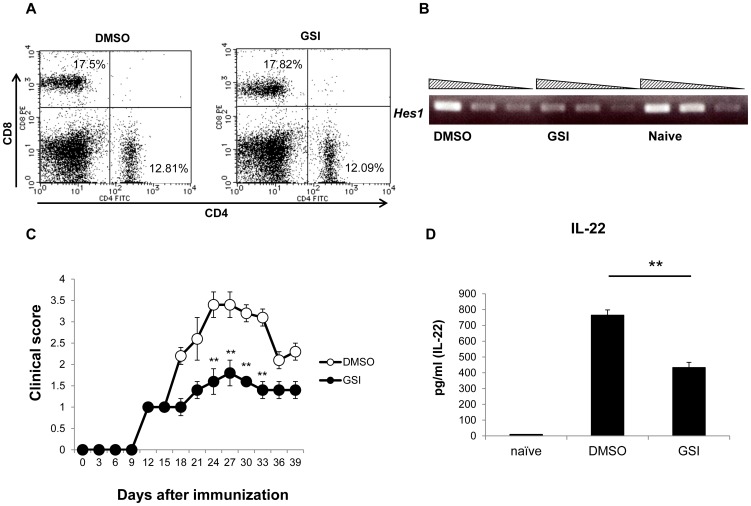
GSI treatment is useful to treat EAU. B6 mice were immunized with IRBP to induce EAU and EAU clinical scores were evaluated until day 39. GSI was administered to the mice according to the following schedules; the day of IRBP immunization is day 0. 2-day intervals (from day 13 to day 39). (A) CD4 and CD8 expression in the draining lymph nodes at day 14 were evaluated by flow cytometry. (B) CD4^+^ T cells were purified from draining lymph nodes of mice that received GSI or DMSO 1 day before and expression of *Hes1* was evaluated by PCR (from left to right; 100 ng, 10 ng, 1 ng of mRNA). (C) EAU clinical scores, (D) CD4^+^ T cells were purified from draining lymph nodes on day 12. Purified CD4^+^ T cells were cultured with irradiated spleen cells from B6 mice in the presence of IRBP protein (50 µg/ml) for 3 days. IL-22 in culture supernatants was evaluated by ELISA. The data are the mean ± SD of triplicate cultures. **p<0.01. The data in this Figure are representative of three independent experiments.

We next examined if a γ-secretase inhibitor that blocks Notch signaling is able to suppress EAU in order to evaluate the potential of Notch modulation as a therapy for EAU. GSI was administered intraperitoneally daily from day 12 to day 18. GSI treatment did not change any mouse activity at a glance or body weight (data not shown), nor did it affect the ratio of CD4^+^ and CD8^+^ T cells (Fig. 5A). GSI treatment reduced the expression of *Hes1*, one of the Notch target genes, compared to the control, indicating that this dose of GSI could inhibit Notch signaling *in vivo* (Fig. 5B). GSI treatment significantly decreased disease progression compared with the control DMSO-treated group (Fig. 5C). CD4^+^ T cells from GSI-treated mice produced lower levels of IL-22 than those from DMSO-treated mice (Fig. 5D). These results indicate that inhibition of Notch signaling by GSI could suppress late-stage EAU.

## Discussion

The present study aimed to investigate the role of Notch signaling in the development of EAU. *Rbpj* deficiency in T cells impaired EAU development by inhibiting IL-22 production from CD4^+^ T cells without affecting IRBP-specific T cell proliferation or Th17 differentiation. IL-22 acted as an inflammatory cytokine during the late phase but not the early phase of EAU progression. The inhibition of Notch signaling by GSI even after EAU was established suppressed EAU clinical scores. These data suggest that the Notch-Rbpj axis regulates the production of IL-22 from CD4^+^ T cells, which is associated with the progression of EAU development.

Previous research has demonstrated that Notch signaling controls the functional differentiation of CD4^+^ T cells [11] [12] [13] [14] [23] [15]. Consistent with our previous study [23], IL-22 production from CD4^+^ T cells after immunizing T cell-specific Rbpj-deficient mice with IRBP was impaired, which was associated with enhanced EAU susceptibility. IL-22 is an IL-10 family cytokine that has pleiotropic roles in inflammatory responses, depending on the disease context [25] [26] [23] [27]. For instance, IL-22 has protective roles in ConA-induced hepatitis [26] [23]. In contrast, IL-22 regulates the development of the skin inflammation pathology seen in psoriasis [27]. The present study showed that administration of rIL-22 during the late phase aggravated EAU in Rbpj^f/f^-CD4 mice, while clinical scores were unaffected when rIL-22 was administered only during the early phase. These data suggest that Notch-mediated IL-22 production has a pathological role only during the late stage of EAU, although we cannot exclude the possibility that the amount of rIL-22 during the early phase is not enough to impact EAU susceptibility. This finding raises the questions of how IL-22 contributes to late-phase EAU pathology without affecting the initial T cell proliferation. A previous report suggested that primary human keratinocytes exposed to IL-22-producing cells expressed a variety of genes involved in innate immune pathways and the induction and modulation of adaptive immunity [28]. These findings suggest that IL-22 may act as a direct inducer for aggravating EAU pathology by interacting with various cells. The second possibility is that IL-22 may have a suppressive effect on the immune inhibitory functions of immune or non-immune cells that are relevant to EAU pathology. In addition, it should be noted that the clinical scores of T cell-specific Rbpj deficient mice that received rIL-22 over the course of EAU could not reach those of control mice, suggesting that Notch signaling in T cells has IL-22 independent pathological roles in the progression of EAU.

A previous study revealed that mice treated with IL-22 generated decreased numbers of IFN-γ^+^ and IL-17^+^ uveitogenic T cells with increased numbers of Foxp3^+^ regulatory T cells, which are induced by CD11b^+^ antigen-presenting cells [29]. This study revealed that rIL-22 treatment attenuates EAU. The discrepancy over the roles of IL-22 in EAU between this study and our present study might be due to the distinct mouse strain used for EAU induction because the past study used B10RIII mice and we used B6 mice. The difference of mouse strain might be attributable to the distinct responses to rIL-22 or a differential requirement of IL-22 for inflammatory responses. Another possibility is the distinct schedule of rIL-22 treatment, because IL-22 has proinflammatory roles or anti-inflammatory roles in different disease contexts [27] [26] [23], which suggests that the roles of IL-22 or the required amount of IL-22 for inhibiting or promoting inflammation might be different during the course of EAU.

It is noteworthy that treatment of EAU with GSI could significantly suppress EAU clinical scores. This treatment with GSI also reduced the production of IL-22, and the suppression of EAU by GSI was reverted by administrating rIL-22 (data not shown). Although Rbpj interacts with p53 [30] and Rbpj-independent Notch signaling was reported [31] [32], these data strongly suggest that Rbpj-Notch pathway is involved in EAU development. These data also suggest that Notch blockade would be useful in treating autoimmune uveitis. However, Notch signaling is crucial for regulating non-immune cells in addition to immune cells. Therefore, it is necessary to determine the concentration of GSI for exerting positive drug effects while avoiding negative side effects. In addition, GSI is not a specific inhibitor of Notch because many other molecules use γ-secretase for signaling. Therefore, we cannot fully exclude the possibility that GSI treatment is also involved in the suppression of a Notch-independent pathway in our study, which might have some role in the suppression of EAU.

In conclusion, the idea that Notch-mediated IL-22 is involved in the progression of late-phase EAU development supports our hypothesis that Notch blockade might be useful as a therapeutic strategy for treating EAU. Indeed, our data in the mouse EAU model indicate that GSI treatment could attenuate EAU even after IRBP immunization. These results highlight the potential usefulness of Notch blockade as a therapy for autoimmune uveitis. Finally, even though EAU progression was prevented by Notch blockade, the question remains of whether memory T cells that differentiate into IL-22-producing T cells would also be blocked under these conditions. Since memory T cells are already present in human ongoing autoimmune uveitis, this situation will also need to be taken into account in clinical settings, and this issue should be analyzed in future studies.
